# Supplementary material for: Paradoxes in leaky microbial trade
Source: Nat Commun. 2017 Nov 8;8:1361. doi: 10.1038/s41467-017-01628-8 (PMC5678203; doi:10.1038/s41467-017-01628-8)
Supplement: Supplementary file 1 — Supplementary Information [file 41467_2017_1628_MOESM1_ESM.pdf]

Supplementary Note 1: Effect of diffusion and degradation rates

In Figures 1 and 2, we illustrate the effects of varying the rates of diffusion of metabolites, D, and degradation of metabolites, μ, relative to the rate at which they are consumed by the growth reaction. (We have taken the reaction rate constant to be 1, setting the units of time). As we have shown in the main text, changing the diffusion coefficient from $D = 0$ to $D = 3$ expands the region of parameter space allowing coexistence from a 1-dimensional curve to a region occupying most of the quadrant where each cell type is more efficient in producing some metabolite. As we can see in Figure 1, this change is gradual as a function of D, and as D tends to infinity, the coexistence region expands to the entire quadrant. Moreover, the region of complete specialization by both cell types expands and also tends to extend to the entire quadrant. By comparison, the changes that occur as a function of the degradation rates are minimal, but a smaller μ does tend to support coexistence and complete specialization.

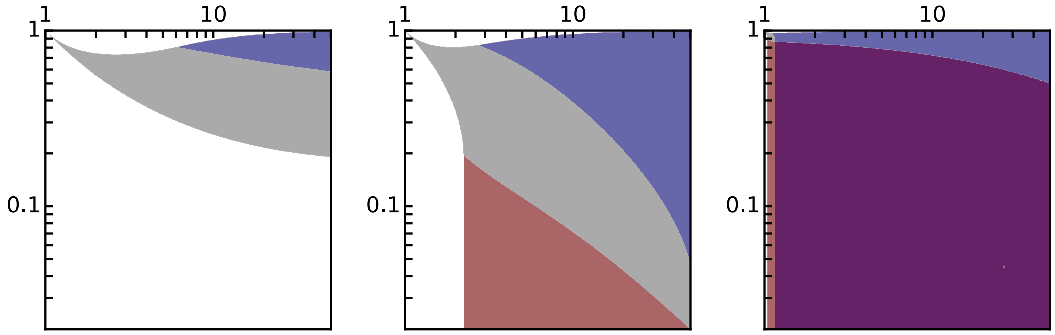

Supplementary Figure 1. Effect of diffusion rate. The region of parameter space allowing coexistence grows as a function of the diffusion rate D. The panels of this figure are analogs of Figure 2b for $D = 0.5$ (left), $D = 1$ (center), and $D = 10$ (right).

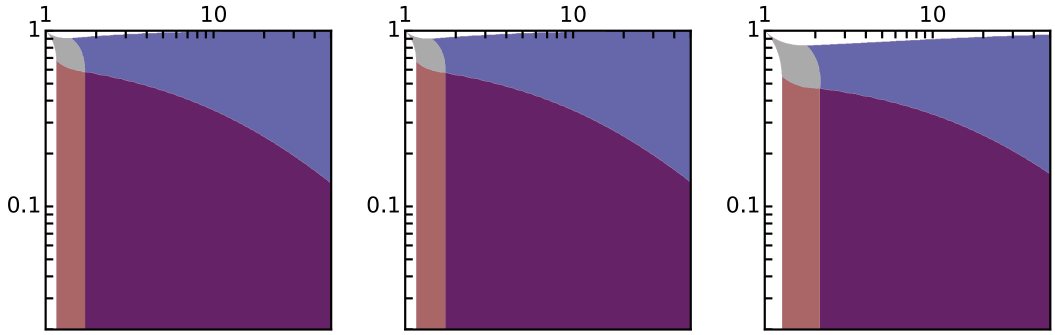

Supplementary Figure 2. Effect of degradation rate. The effect of the degradation rate μ is minimal. The panels of this figure are analogs of Figure 2b for $\mu = 0.02$ (left), $\mu = 0.1$ (center), and $\mu = 1$ (right).

At smaller diffusion coefficient, an interesting behavior of the model becomes more pronounced in the context of the cursed of decreased inefficiency. For certain regions of parameters, we observe two noncontiguous regions of decreasing growth rate in response to decreased inefficiency (see Figure 3). They are associated with the successive transitions of the two cell types from complete specialization to lower specialization.

Supplementary Note 2: Effect of response speed

In the main text, we use the simple assumption that cells' production decisions can be regulated on a much shorter time scale than the population dynamic time scale. While this assumption is convenient, it

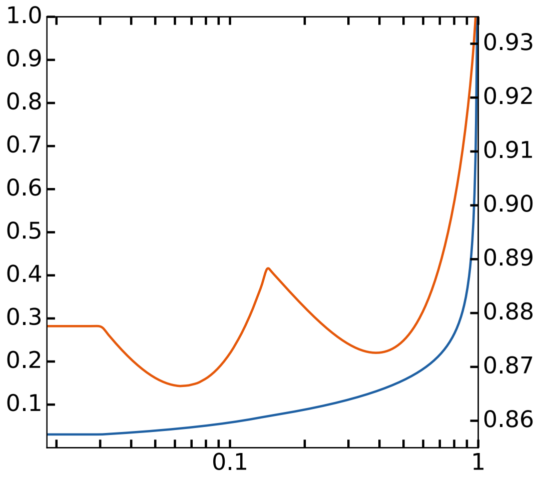

Supplementary Figure 3. **The curse of decreased inefficiency redux.** In some cases, our model predicts two noncontiguous regions of decreasing growth rate as a function of increased efficiency. Here we show the analog of Figure 4b for the case where $D = 1$, $a_{A,1} = a_{B,1} = 1$, $a_{B,2} = 30$, and $a_{A,2}$ varies.

is not necessary: even if the production decisions move toward their optimal value at a rate much slower than the population growth rate, say through small and rare mutations, it is still the case that the only equilibrium situation is the same equilibrium we identify in the main text.

We illustrate this point using two different response models. First, consider the following dynamical system, where the response is toward the optimal value of the production budget allocation, but gradual instead of instantaneous:

$$\begin{aligned} \frac{dp_{X,i}}{dt} &= \beta(p^*_{X,i} - p_{X,i}), \qquad \text{for } i = 1,2, \quad X = A,B \\ \frac{dn_1}{dt} &= n_1(1-n_1)(g_1 - g_2). \end{aligned} \tag{S1}$$

Here, $p^*_{X,i}$ is the optimal production decision for cell type i in the present environment, g_i is the growth rate of cell type i under the present frequencies and production decisions, and β is a parameter determining the relative speed at which production decisions adjust toward the optimum. In Figure 4, we show trajectories of this system for $\beta = 0.05$, starting at different initial relative frequencies. Even with the lag between population dynamics and production decisions, we find that trajectories converge to the same equilibrium as found in our original analyses.

Second, we consider a model where production decisions are fixed at a genetic level and adjust only as a result of mutations that provide a fitness advantage and fix in the cell type population. At regular intervals throughout the evolution of the population, we pick one of the cell types with probability proportional to its frequency in the population and introduce a mutated strain with a randomly drawn production budget allocation. We show a sample of the dynamics associated with this model in Figure 5. We find that after some time the strains representing the equilibrium production allocation found in the original analysis take over their respective cell type populations and the cell types reach their equilibrium relative frequencies. This situation is in equilibrium because no new mutated strain can invade its cell type population, which is already at the optimal production allocation for the current environment.

Supplementary Note 3: Effect of different forms of the model

Effect of unequal base growth rates

In the main text, we only consider situations where the two cell types have identical growth functions. We found that whenever one cell type was more efficient at both production tasks, coexistence in equilibrium was impossible, and the more efficient cell type took over. Also, we found that when cell types stably coexist, both cell types grow faster compared to if they were isolated.

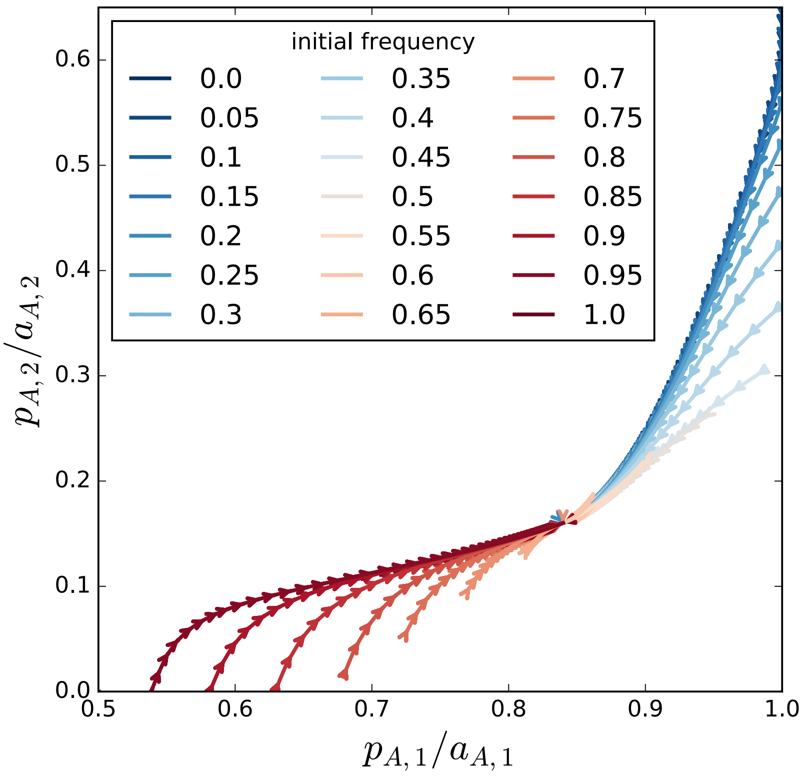

Supplementary Figure 4. Effect of slower response. Trajectories of solutions to Eq. (S1) starting from the Nash equilibrium at some non-equilibrium relative frequency. The horizontal (vertical) axis shows the fraction of the budget devoted by cells of type 1 (2) to producing A. The production efficiencies used are $a_{A,1} = a_{B,1} = 1$, $a_{A,2} = 0.67$, and $a_{B,2} = 1.49$, and the response speed parameter used is $\beta = 0.05$. All trajectories arrive at the same equilibrium as found when the time scale for production decisions is much faster than population dynamics.

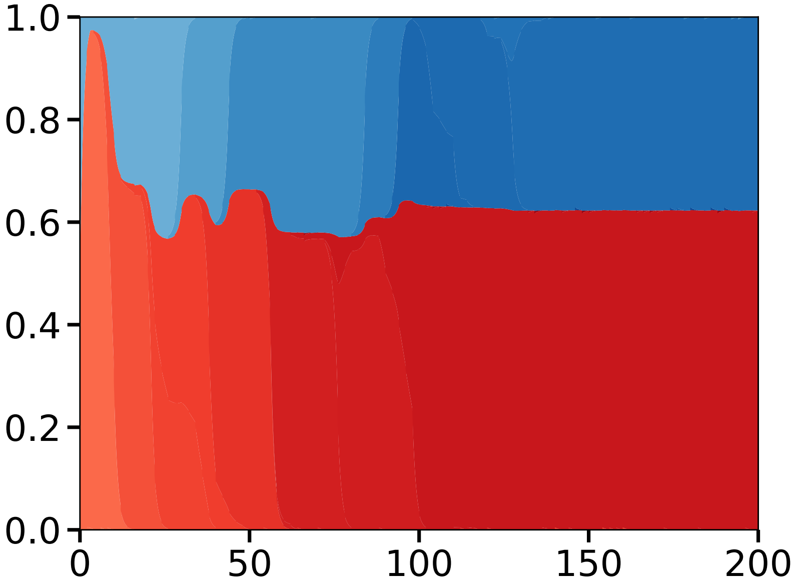

Supplementary Figure 5. Simulation of mutation dynamics. Relative frequency in the population of cells of type 1 (red) and type 2 (blue) where strains with different production allocations are indicated with different shades, where brighter shades correspond to more specialized production toward the comparative advantage metabolite for the cell type. The production efficiencies used are $a_{A,1} = a_{B,1} = 1$, $a_{A,2} = 0.67$, and $a_{B,2} = 1.49$. The horizontal axis corresponds to time renormalized by the difference in growth rate between the fastest growing strain and the slowest in order to display the full range of population dynamics. We see interesting behavior, such as when a strain of cell type 1 takes over that cell type population but in doing so reduces that cell type's relative frequency, or when the dominant strain of cell type 2 goes from being a nonspecializing strain to a highly specializing strain and back to a moderately specializing strain. However, we find that the eventual equilibrium is the same as when production decisions are regulated at a fast time scale.

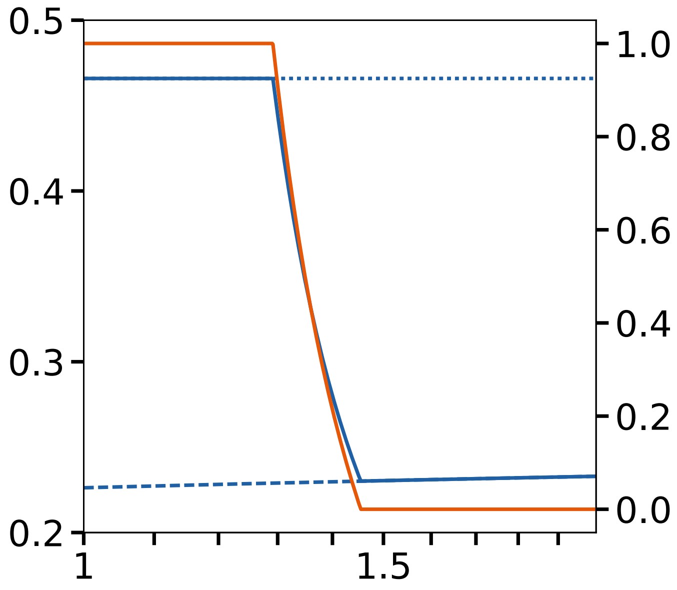

Supplementary Figure 6. Effect of unequal base growth rates. Growth rate (blue) and relative frequency of type 1 (orange) in equilibrium as a function of the ratio of rate constants in the growth function of the two cell types, k_2/k_1. Type 1 has higher efficiencies, $a_{A,1} = a_{B,1} = 1$, than type 2, $a_{A,2} = a_{B,2} = 1/2$, at both production tasks, but grows at a lower rate under the same metabolite concentrations. When the difference in base growth rate is small, cell type 1 takes over the population due to its higher efficiency, and when the difference is large, cell type 2 takes over due to its faster growth. In an intermediate range, the two types coexist, and grow at a rate that is faster than what type 2 in isolation would achieve (dashed line), but slower than what type 1 in isolation would achieve (dotted line).

By contrast, here we consider an example where $a_{A,1} > a_{A,2}$ and $a_{B,1} > a_{B,2}$, but $g_i = k_i A_i B_i$ and $k_2 > k_1$. As a function of the ratio of the rate constants k_2/k_1, we show the resulting equilibrium growth rate and relative frequencies in Figure 6. We find that coexistence does occur over a range of ratios and that cells of type 1 have a higher growth rate in isolation than in this coexistence.

Effect of different functional form for the growth function

In the main text, we use a growth rate function corresponding to an elementary reaction of two reagents. We found that there is always a unique Nash equilibrium for any relative frequency of the two cell types, and a unique relative frequency where the growth rates at the Nash equilibrium match (or the degenerate solution, where one type always has a higher growth rate and drives the other type extinct). Here we considered the effect of using a different growth function, corresponding to Michaelis-Menten, or Monod, kinetics:

$$g_i = \frac{A_i K_{A,i}}{A_i + K_{A,i}} \frac{B_i K_{B,i}}{B_i + K_{B,i}}. \tag{S2}$$

We find (see Figure 7) that the saturation of the growth rate with increased concentration of the metabolites does not dramatically affect the range of relative efficiencies where we observe coexistence, specializing, and the paradoxical behaviors we highlight in the main text. However, we find that it is no longer the case that the Nash equilibrium is unique, and that bistability is possible in certain regions of relative efficiencies.

Effect of soft production constraint

In the main text, we model the limits to production of metabolites as a budget constraint. In some cases it is more appropriate to consider metabolite production as a direct trade-off with growth. Therefore, instead of a hard budget constraint $P_i(p_{A,i}, p_{B,i}) \leq P_{\max}$, we consider a soft constraint affecting the growth

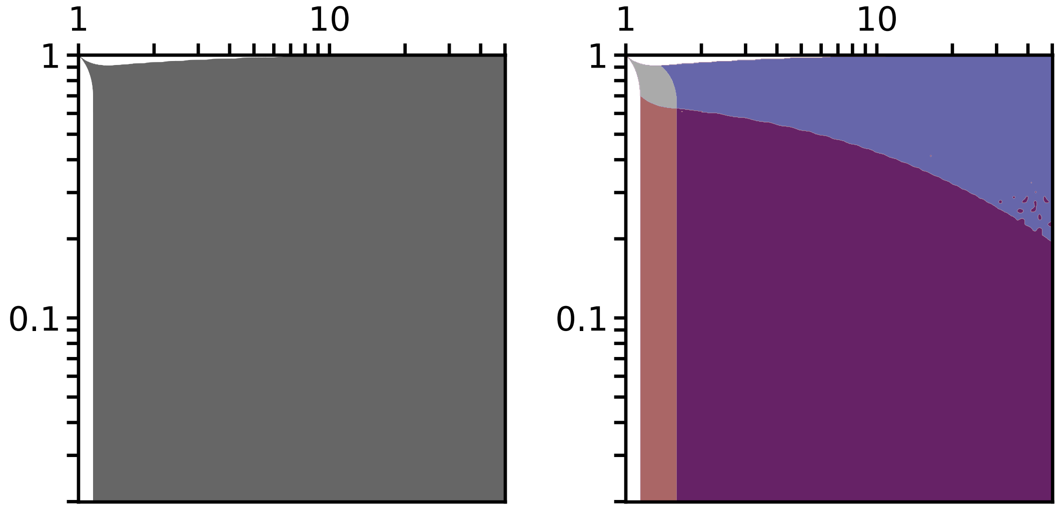

Supplementary Figure 7. Effect of different functional form for the growth function. Using a growth function corresponding to Monod kinetics (Eq. (S2)), with $K_{A,i} = K_{B_i} = 2$ for $i = 1,2$, we find the resulting equilibrium. In the region of high efficiency of type 2 in producing B, around where type 2 shifts towards complete specialization, we find nonuniqueness of the Nash equilibrium and more than one stable relative frequency. This behavior is evident in the right-hand plot, where two equilibria are found alternately, only one of which has cells of type 2 completely specialized.

rate: $g_i = g_i^{(\text{base})}(A_i, B_i) - P_i(p_{A,i}, p_{B,i})$, where $g_i^{(\text{base})}(A_i, B_i)$ is a base growth rate that depends on the metabolite concentrations, and $P_i(p_{A,i}, p_{B,i})$ is the reduction in growth rate due to devoting resources to metabolite production.

To prevent the optimization of growth rate from being unbounded, we use the Monod growth function Eq. (S2) for the base growth rate. The simplest choice for the penalty function is a linear form, $P_i(p_{A,i}, p_{B,i}) = c_{A,i}p_{A,i} + c_{B,i}p_{B,i}$, where each of the metabolites carries an independent cost in terms of growth rate. Using these forms we find that paradox 1 (the curse of increased efficiency) remains in effect over a large region of the production efficiencies, but that paradox 2 (the curse of decreased inefficiency) no longer appears. We associate this finding with the fact that with a linear soft constraint, there is no longer a trade-off for cells of type 2 between producing metabolite B and producing metabolite A when it becomes less inefficient at producing the latter. That is, there is no pressure to decrease production of B in order to increase production of A since their costs are independent. This interpretation is bolstered by the fact that we do observe paradox 2, as we show in Figure 8, when we use a quadratic soft constraint,

$$P_i(p_{A,i}, p_{B,i}) = (c_{A,i}p_{A,i} + c_{B,i}p_{B,i})^2. \tag{S3}$$

Reaction-diffusion model with explicit extracellular concentrations

In the main text, we present a dynamical model of reaction-diffusion dynamics (Eq. (2)) that includes only the intracellular concentrations inside the two cell types and treats diffusion as occurring directly between cells, and not mediated by an extracellular environment. Here we show that this model follows as a simplification of a more explicit model that includes an extracellular environment that mediates diffusion:

$$\begin{aligned}
\frac{dA_i}{dt} &= p_{A,i} + D(A_{\text{ext}} - A_i) - \mu A_i - s_{A,i}g_i(A_i, B_i) \\
\frac{dB_i}{dt} &= p_{B,i} + D(B_{\text{ext}} - B_i) - \mu B_i - s_{B,i}g_i(A_i, B_i) \\
V_{\text{ext}}\frac{dA_{\text{ext}}}{dt} &= N_1 vD(A_1 - A_{\text{ext}}) + N_2 vD(A_2 - A_{\text{ext}}) \\
V_{\text{ext}}\frac{dB_{\text{ext}}}{dt} &= N_1 vD(B_1 - B_{\text{ext}}) + N_2 vD(B_2 - B_{\text{ext}}).
\end{aligned} \tag{S4}$$

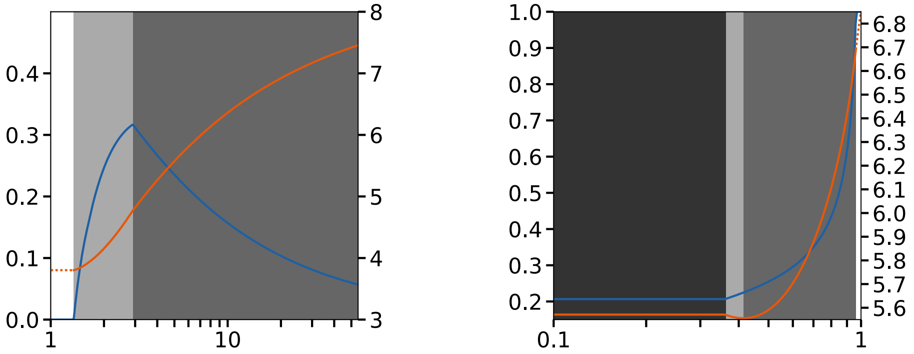

Supplementary Figure 8. Effect of soft production constraint. When we use a Monod growth function (Eq. (S2)) with a soft production constraint (growth penalty) of a quadratic form (Eq. (S3)), we observe paradox 1 persisting much as it did in the model considered in the main text (left). Paradox 2 is diminished in the magnitude of its effect and the region of its applicability, but is still observed (right).

Here v is the average volume of a cell and V_{ext} is the volume of the external medium. In a steady state of this system of ODEs, we have $A_{\text{ext}} = (N_1A_1 + N_2A_2)/(N_1 + N_2)$ and $B_{\text{ext}} = (N_1A_1 + N_2B_2)/(N_1 + N_2)$. So, if we are only interested in steady states of this system of ODEs we can make this substitution to arrive at the equations (2). Throughout the paper, we are assuming that the reaction-diffusion dynamics proceed so quickly compared to the other processes in the system that they are always in a steady state. Therefore, instead of the ODEs above, we are actually working with the algebraic equations

$$\begin{aligned} 0 &= p_{A,i} + D(A_{\text{ext}} - A_i) - \mu A_i - s_{A,i} g_i(A_i, B_i) \\ 0 &= p_{B,i} + D(B_{\text{ext}} - B_i) - \mu B_i - s_{B,i} g_i(A_i, B_i) \\ A_{\text{ext}} &= (N_1A_1 + N_2A_2)/(N_1 + N_2) \\ B_{\text{ext}} &= (N_1A_1 + N_2B_2)/(N_1 + N_2). \end{aligned} \tag{S5}$$

When the steady state external concentration is substituted into the equations for the intracellular concentrations, we recover the unmediated-diffusion model used in the main text, and it is clear that the steady states of the two models are identical.

Supplementary Figure: Paradox 3, timescale of growth/relative frequency tradeoff

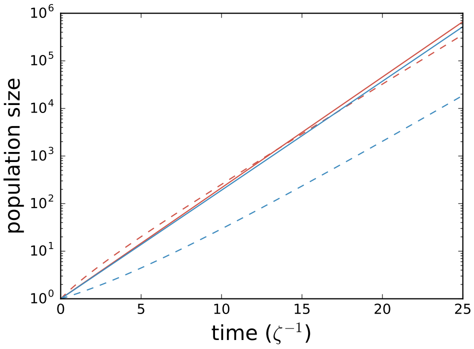

Supplementary Figure 9. Growth trajectories for Paradox 3. Population size as a function of time for cell type 1 (red) and 2 (blue) when each cell type maximizes its own growth rate (solid) and when both cell types maximize the growth rate of cell type 1 (dashed). The population of cells of type 1 in the competitive scenario overtakes that in the control scenario after it grows by a factor of 1420.

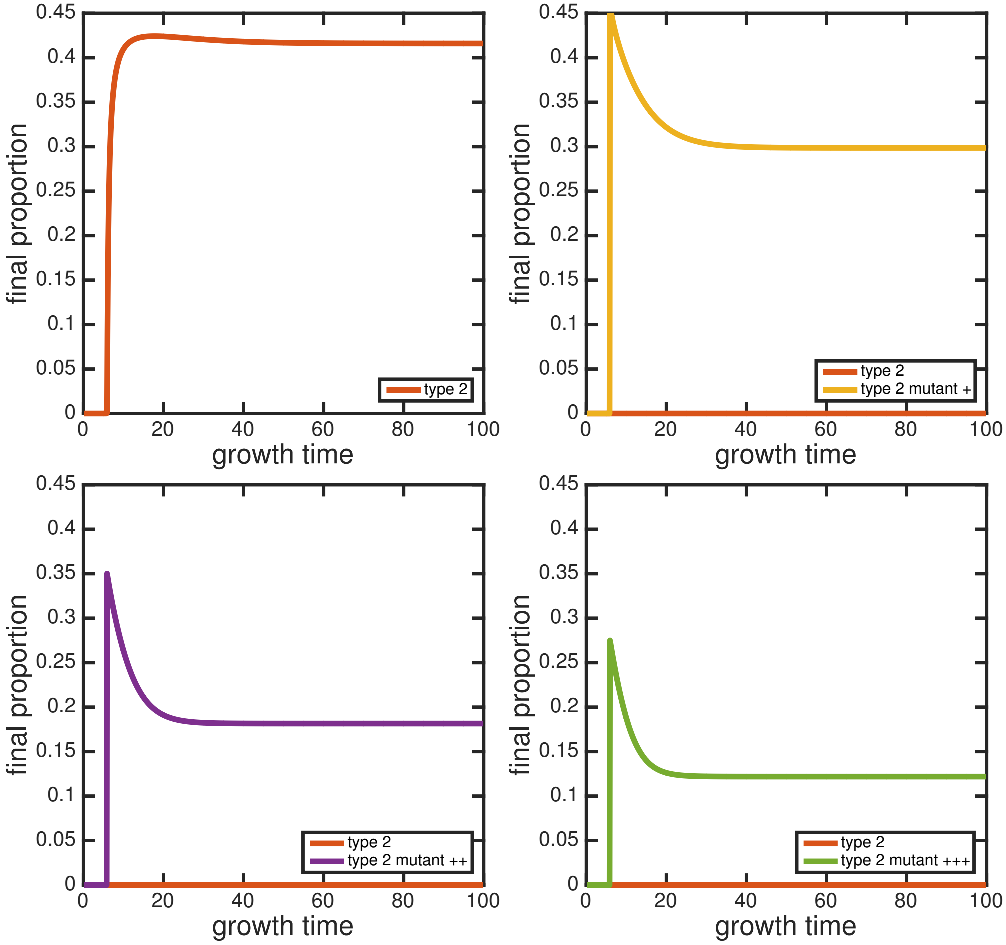

Supplementary Figure 10. Effect of different durations in patches for paradox 1. Each plot shows the final relative frequencies of cell types as a function of the duration of growth within a patch. The top left shows the relative frequency of type 2 cells without any mutants. The other plots show the results when a type 2 mutant invades. We find that as long as the ancestral type 2 can coexist with type 1 cells, in all cases the mutants invade and replace the ancestral type 2 cell. Moreover, they end up at lower relative frequency than the ancestral type (except for a small range of growth times for the + mutant).

Supplementary Note 4: Duration in patch

We explore the effects of changing the duration of growth within patches in our evolutionary metapopulation model. For the case corresponding to paradox 1 in which mutants have an increased efficiency in producing the B metabolite, we find that the duration within the patch has little effect on observing the phenomenon (see Figure 10). As long as type 2 cells can coexist with type 1 cells, we find that type 2 mutants can invade and replace the ancestral type. In all cases, except for a narrow range for the + mutant, we find that the invader ends up at a lower relative frequency than the type it replaced.

The situation is very different in the scenario pertaining to paradox 2. Because the mutants with increased efficiency in making the A metabolite produce mixed populations with a lower growth rate, it is difficult for them to invade. Indeed, the range of growth durations within a patch that permit a successful invasion is more narrow than in the case of paradox 1 (see Figure 11). If we consider the opposite scenario where type 2 cells that are less efficient in making A invade a more efficient mutant populations, we find that this can occur over a large range of growth durations (see Figure 12).

Supplementary Note 5: Paradox 3, invasion of the manipulator

We consider the effects of growth duration in a patch and initial relative frequency on the success of an invading type 2 manipulator. We use the parameters from Figure 5 in the main text where Paradox

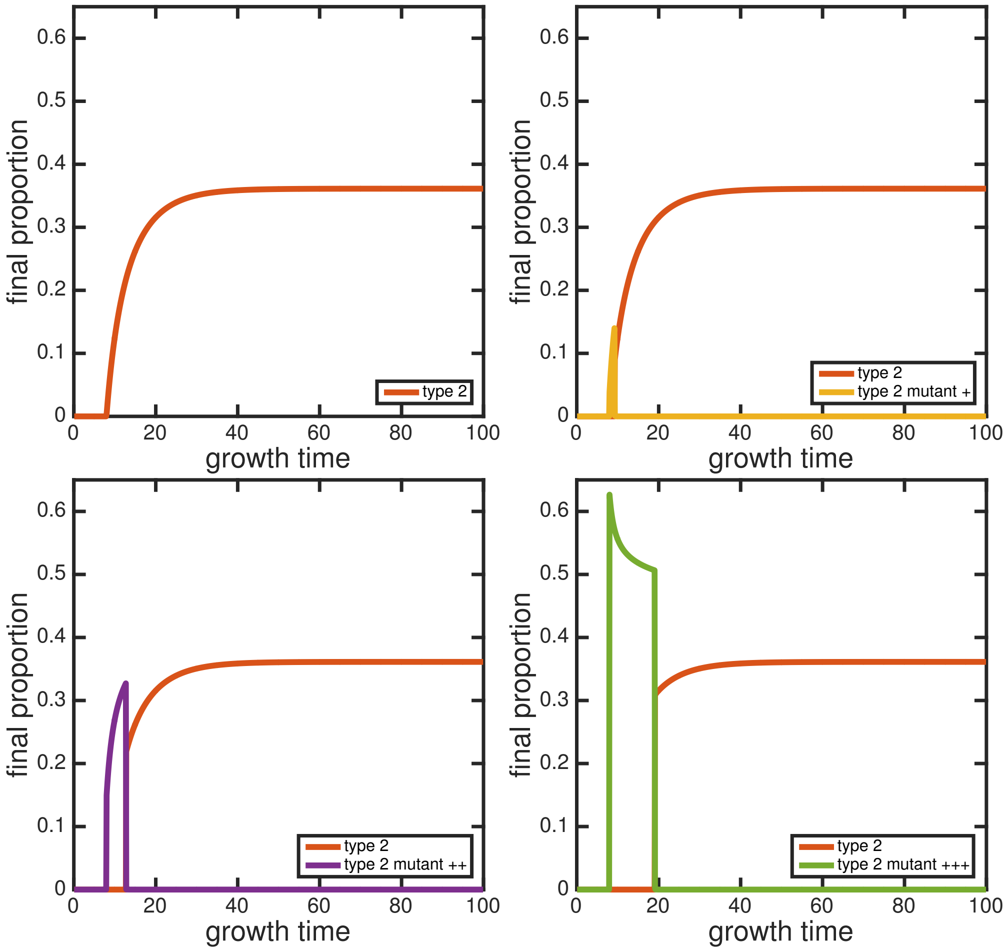

Supplementary Figure 11. Effect of different durations in patches for paradox 2. The same as Figure 10 except corresponding to paradox 2 where mutants have increased efficiency in making the *A* metabolite. There is only a narrow range of growth duration in a patch in which mutants can invade. This is because patches with type 1 and type 2 ancestral cells outproduce patches with type 1 and type 2 mutant cells.

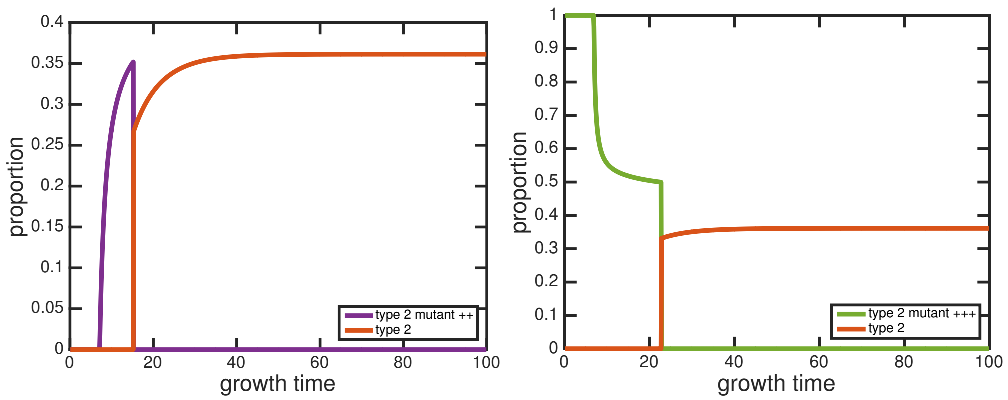

Supplementary Figure 12. Invasion of mutant populations by less efficient type 2 cells. This is the inverse plot of Figure 11 in which we consider the ancestral type 2 cell invading a mutant population. We find that once the growth duration in a patch is long enough, the less efficient ancestral type can invade the more efficient mutant population. Again, this is because patches with type 1 and type 2 ancestral cells outproduce patches with type 1 and type 2 mutant cells.

3 is discussed so that $a_{A,1} = a_{B,1} = 1$, $a_{A,2} = 0.67$, and $a_{B,2} = 1.49$. Figure 13 shows that even though manipulator can control the production of both type 1 and type 2 cells, it cannot invade from low relative frequency regardless of the duration in a patch. Interestingly, if it starts at a higher fraction of the population it can invade but drives the other cell types extinct.

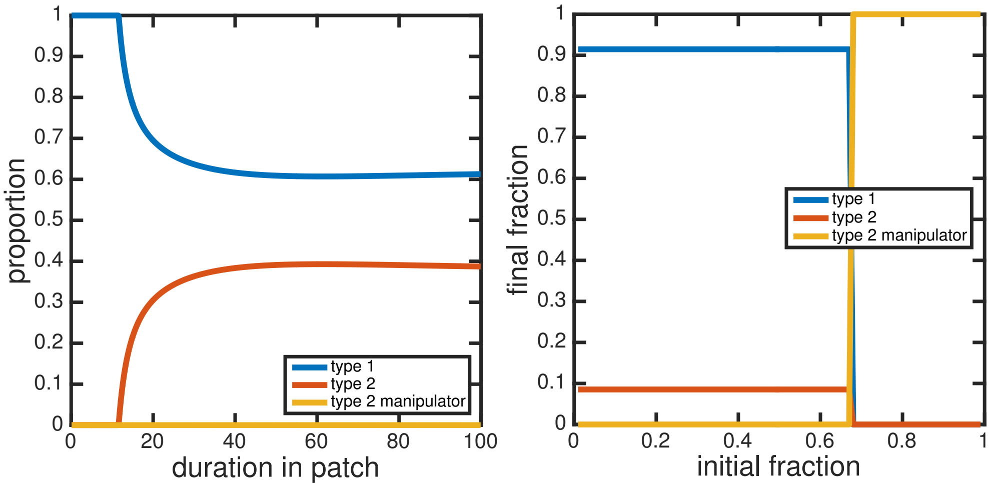

Supplementary Figure 13. **Invasion of the manipulation**. (Left) The final relative frequencies of cell types is shown as a function of the duration of growth in a patch. We find that the manipulator can not invade from low frequency regardless of the duration of the patch. (Right) The final relative frequencies of cell types is shown as a function of the initial fraction of the invading type, for a patch duration of 12.5. We find that if the initial fraction of the manipulator is high enough it can invade but in so doing it drives the other cell types extinct.
